# Therapeutic Effects of Combined 6-Shogaol and Ibudilast on Neuroinflammation and Behavioral Deficits in a Cuprizone Mouse Model of Multiple Sclerosis

**DOI:** 10.3390/ph19071004

**Published:** 2026-06-28

**Authors:** Gadah Ali Alshahrany, Kholoud A. Alyami, Noor Ahmed Alzahrani, Mohammad Zubair Alam, Badrah S. Alghamdi, Ulfat M. Omar, Abeer A. Banjabi, Huda F. Alshaibi, Rana Jamalaldin Jambi, Kholoud M. Al-Otaibi, Hadeil M. Alsufiani

**Affiliations:** 1 Department of Biochemistry, Faculty of Sciences, King Abdulaziz University, Jeddah 21589, Saudi Arabia; ksalehalyami@stu.kau.edu.sa (K.A.A.); nsalahalzahrani@stu.kau.edu.sa (N.A.A.); uomer@kau.edu.sa (U.M.O.); abanjabi@kau.edu.sa (A.A.B.); halshaibi@kau.edu.sa (H.F.A.); halsufiani@kau.edu.sa (H.M.A.); 2 Neuroscience and Geroscience Research Unit, King Fahd Medical Research Center, King Abdulaziz University, Jeddah 21589, Saudi Arabia; mzalam@kau.edu.sa (M.Z.A.); basalghamdi@kau.edu.sa (B.S.A.); 3 Department of Medical Laboratory Sciences, Faculty of Applied Medical Sciences, King Abdulaziz University, Jeddah 21589, Saudi Arabia; 4 Department of Physiology, Faculty of Medicine, King Abdulaziz University, Jeddah 21589, Saudi Arabia; 5 Princess Dr. Najla Bint Saud Al-Saud Center for Excellence Research in Biotechnology, King Abdulaziz University, Jeddah 21589, Saudi Arabia; 6 Stem Cell Unit, King Fahd Medical Research Center, King Abdulaziz University, Jeddah 21589, Saudi Arabia; 7 The Applied College, Department of Health Information Technology, King Abdulaziz University, Jeddah 21589, Saudi Arabia; ranajambi8@gmail.com; 8 Department of Chemistry, Faculty of Science, Al-Baha University, Al-Baha 65799, Saudi Arabia; khalroqi@bu.edu.sa; 9 Experimental Biochemistry Unit, King Fahd Medical Research Center, King Abdulaziz University, Jeddah 21589, Saudi Arabia

**Keywords:** multiple sclerosis, cuprizone, combination therapy, ibudilast, 6-shogaol, neuroinflammation, natural products

## Abstract

**Background/Objectives**: Multiple sclerosis (MS) is a chronic autoimmune disease of the central nervous system characterized by inflammation, demyelination, and axonal loss. Despite available therapies, there is currently no effective cure for MS. Ibudilast (IBD), a phosphodiesterase inhibitor, and 6-shogaol (SH), a bioactive compound from ginger, have independently shown therapeutic potential in MS models. This study aimed to evaluate the therapeutic efficacy of combining SH and IBD in modulating neuroinflammation and improving functional recovery in a cuprizone (CPZ) mouse model of MS. **Methods**: Male SWR/J mice were exposed to 0.3% CPZ for 5 weeks to induce demyelination, followed by 4 weeks of spontaneous remyelination after CPZ withdrawal. During remyelination, the CPZ group was subdivided into four groups: no therapy, SH (25 mg/kg), IBD (10 mg/kg), and SH + IBD. Behavioral tests were used to assess locomotion, muscle strength, coordination, and memory. Gene expression of proinflammatory and anti-inflammatory cytokines was analyzed in brain tissue. **Results**: The combined treatment significantly improved locomotor activity, muscle strength, and memory during remyelination phases while suppressing proinflammatory gene expression and enhancing anti-inflammatory pathways in the brain. **Conclusions:** SH and IBD combination therapy provides enhanced anti-inflammatory and functional benefits compared with monotherapies, supporting its potential as a promising multi-target therapeutic strategy for improving functional recovery and modulating neuroinflammation during the spontaneous remyelination phase following CPZ withdrawal.

## 1. Introduction

Multiple sclerosis (MS) is a long-term immune-mediated disorder affecting the central nervous system (CNS), in which inflammatory processes lead to damage of the myelin sheath and, over time, injury to axons [1]. Although the precise etiology of MS has not been fully established, current evidence suggests that disease susceptibility results from a complex interaction between genetic predisposition and environmental exposures [2]. Globally, more than 2.8 million individuals are estimated to be living with MS, with rising prevalence reported in the Middle East and Saudi Arabia [3,4]. Clinically, MS presents with a wide range of neurological deficits, including motor impairment and cognitive decline, which significantly reduce the quality of life [2,5].

Neuroinflammation plays a central role in the pathogenesis of MS. Demyelination in MS results from immune-mediated destruction of the myelin sheath, primarily by T cells, B cells, and macrophages, which release proinflammatory cytokines such as tumor necrosis factor-alpha(TNF-α), interleukin-6 (IL-6), and interleukin-1 beta (IL-1β), leading to axonal injury and neuronal dysfunction [6]. In parallel, cyclooxygenase-2 (COX-2) is upregulated downstream of these inflammatory pathways, amplifying prostaglandin-mediated neuroinflammation and exacerbating neural damage [7]. Conversely, anti-inflammatory cytokines such as interleukin-4 (IL-4) and the transcription factor nuclear factor erythroid 2–related factor 2 (Nrf2) play crucial yet distinct roles in mitigating neuroinflammation, reducing oxidative stress, and promoting tissue repair. IL-4 promotes a shift toward the M2 microglial phenotype, supporting tissue recovery, whereas Nrf2 activates antioxidant response elements and suppresses nuclear factor kappa-light-chain-enhancer of activated B cells (NF-κB )dependent inflammatory signaling. In contrast, NF-κB functions as a central proinflammatory regulator by amplifying inflammatory cascades and upregulating cytokines such as TNF-α, IL-1β, and COX-2, thereby sustaining glial activation and contributing to neuronal injury [8,9,10]. Persistent myelin debris and chronic glial activation can impede oligodendrocyte progenitor cell (OPC) maturation, emphasizing the need for therapeutic strategies that restore the balance between inflammatory and antioxidant signaling to promote myelin repair [6]. Although spontaneous remyelination can occur during early MS, it is often incomplete and insufficient to fully restore neurological function; thus, enhancing endogenous remyelination has become a primary therapeutic goal [6,11].

**Figure 1:**
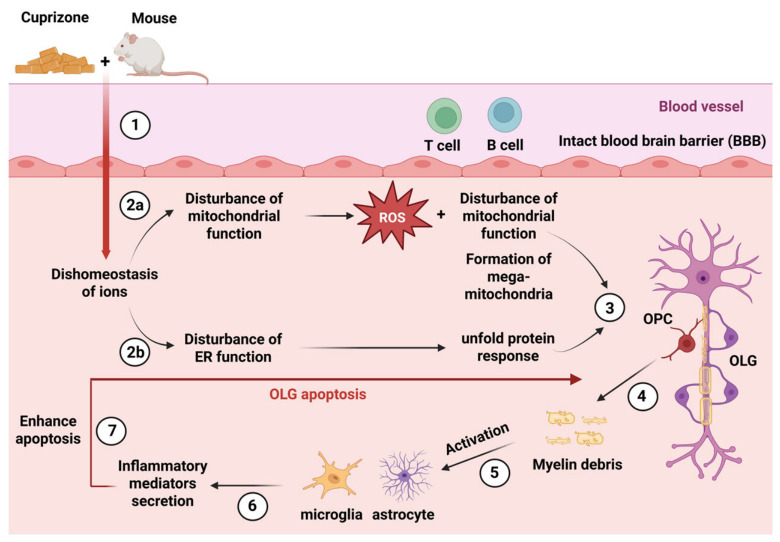
Schematic Overview of the Proposed Mechanism Underlying CPZ Intoxication. Following the oral intake of CPZ by rodents, the compound disrupts the normal functions of the endoplasmic reticulum (ER) and mitochondria, resulting in the death of oligodendrocytes (OLGs) and the buildup of myelin debris. This debris triggers the activation of glial cells, such as microglia and astrocytes, which then release inflammatory molecules that exacerbate the degeneration of OLGs. The entry of B and T cells from the peripheral bloodstream into the central nervous system (CNS) is restricted by the intact blood–brain barrier (BBB). CPZ: cuprizone; OPC: oligodendrocyte progenitor cells. Created in BioRender. ALSHAHRANY, G. (2026) https://BioRender.com/j64xpxn (accessed on 23 June 2026).

To better understand the inflammatory and demyelinating processes observed in MS, experimental animal models have been developed. Among these, the cuprizone (CPZ) model is one of the most widely used nonautoimmune paradigms for studying myelin injury and repair in vivo. The administration of CPZ, a copper-chelating agent, in rodent chow induces selective oligodendrocyte death and reproducible demyelination, particularly within the corpus callosum, accompanied by motor and cognitive deficits that parallel the functional impairments observed in MS patients [12,13]. Figure 1 illustrates key pathological mechanisms in CPZ-induced demyelination, including ion dysregulation, mitochondrial dysfunction, and the activation of glial inflammatory mediators [14]. The CPZ model offers a distinct advantage over autoimmune models such as experimental autoimmune encephalomyelitis (EAE), as the blood–brain barrier (BBB) remains largely intact, making this model ideal for studying demyelination and remyelination in the CNS without involving adaptive immune cells [15,16]. Moreover, spontaneous remyelination occurs following CPZ withdrawal, making this model highly suitable for evaluating pharmacological agents that accelerate or enhance myelin repair [16].

Current treatments for MS primarily aim to suppress inflammation, manage symptoms, and slow disease progression. However, there is still no cure for MS, and ongoing research seeks therapeutic strategies that not only reduce neuroinflammation but also support functional recovery [17]. One promising agent is Ibudilast (IBD), a phosphodiesterase (PDE) inhibitor that exerts potent anti-inflammatory and neuroprotective effects by suppressing glial activation, reducing proinflammatory cytokine release, and crossing the BBB to modulate cyclic nucleotide signaling. Preclinical studies in experimental autoimmune encephalomyelitis (EAE) and CPZ models have shown that IBD reduces neuroinflammation and protects against demyelination [18]. In the phase II SPRINT-MS clinical trial, IBD significantly reduced the rate of brain atrophy in patients with progressive MS, reinforcing its therapeutic potential as a neuroprotective agent [19,20].

Additionally, 6-shogaol (SH), a bioactive phenolic compound derived from Zingiber officinale (ginger), exhibits strong anti-inflammatory and antioxidant properties by inhibiting NF-κB signaling and activating the Nrf2/HO-1 pathway, thereby reducing oxidative stress [21]. Preclinical studies further indicate that SH supports oligodendroglial responses and improves functional outcomes in experimental demyelination models [22,23]. From a pharmaceutical perspective, combining a natural bioactive compound with a clinically investigated neuroprotective drug represents a rational multi-target therapeutic strategy aimed at simultaneously modulating neuroinflammation and oxidative stress, two key contributors to MS pathology. Given their complementary mechanisms, IBD and SH may collectively modulate neuroinflammation and support functional recovery, suggesting a promising functional additive therapeutic strategy for MS.

Despite significant progress in MS research, substantial gaps remain regarding combination therapies that target multiple pathological pathways simultaneously. Most studies have focused on either inflammation modulation or myelin repair. Therefore, this study aimed to investigate the therapeutic potential of a novel combination of IBD and SH on neuroinflammation and functional recovery in a CPZ mouse model of MS. This investigation included behavioral assessments to evaluate motor function, balance, grip strength, and cognitive performance, as well as quantitative PCR analysis to measure the expression levels of key proinflammatory and anti-inflammatory cytokines.

## 2. Results

### 2.1. Body Weight Assessment

**Figure 2:**
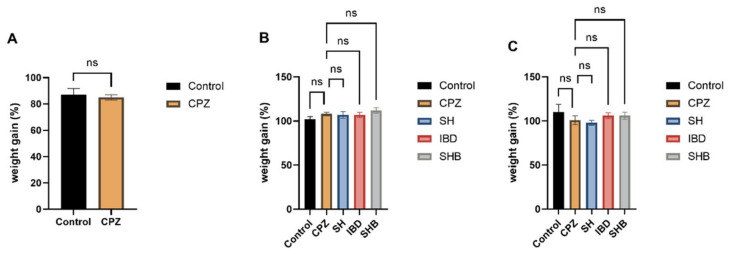
Body weight changes (%) of mice. (**A**) Effects of CPZ on body weight changes (%) during the demyelination stage. (**B**) Effects of SH, IBD, and their combination on body weight changes (%) during early and (**C**) late remyelination stages. Data are presented as mean ± SEM. An unpaired *t*-test was used in demyelination stage (**A**), and one-way ANOVA followed by Tukey’s test was used in early and late remyelination stages ((**B**) and (**C**), respectively). CPZ, cuprizone; SH, 6-shogaol; IBD, Ibudilast; SHB, 6-shogaol + Ibudilast; SEM, standard error of the mean; ANOVA, analysis of variance; ns, nonsignificant.

The overall weight gain results are summarized in Figure 2. There were no significant differences in weight gain percentage between the control and CPZ groups across the demyelination, early, and late remyelination stages (*p* = 0.6756, *p* = 0.6367, and *p* = 0.7540, respectively; Figure 2A–C). Similarly, compared with the CPZ treatment, the SH, IBD, and SHB treatments did not significantly affect weight gain at any stage as shown in Figure 2B,C.

### 2.2. Behavioral Tests

#### 2.2.1. Open Field Test

**Figure 3:**
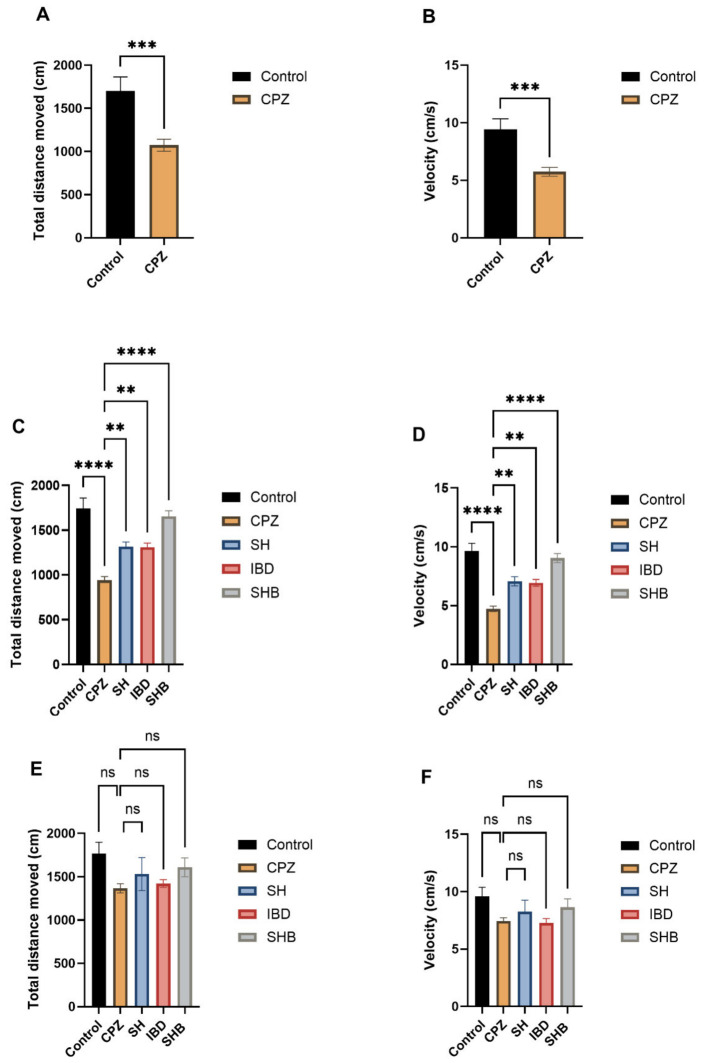
Locomotor activity assessment in the open field test. (**A**) Effect of CPZ on TDM during the demyelination stage. (**B**) Effect of CPZ on velocity during the demyelination stage. (**C**) Effects of different treatments on TDM during the early remyelination stage. (**D**) Effects of different treatments on velocity during the early remyelination stage. (**E**) Effects of different treatments on the TDM during the late remyelination stage. (**F**) Effects of different treatments on the velocity during the late remyelination stage. Data are presented as mean ± SEM; *t*-tests were used in (**A**,**B**), and one-way ANOVA followed by Tukey’s test was used in (**C**–**F**). ** *p* < 0.01, *** *p* < 0.001, **** *p* < 0.0001. CPZ: cuprizone; TDM: total distance moved; SH: 6-shogaol; IBD: Ibudilast; SHB: 6-shogaol + Ibudilast; ns: not significant.

Compared with the control, CPZ significantly reduced TDM and velocity during demyelination (*p* < 0.0001; Figure 3A,B) and early remyelination (*p* < 0.0001). Compared with the CPZ treatment, the SH, IBD, and SHB treatments significantly improved both parameters during early remyelination [TDM: SH (*p* = 0.0042), IBD (*p* = 0.0051), and SHB (*p* < 0.0001); velocity: SH (*p* = 0.0023), IBD (*p* = 0.0044), and SHB (*p* < 0.0001); Figure 3C,D]. No significant differences were observed among the groups during late remyelination (Figure 3E,F).

#### 2.2.2. Rotarod Test

**Figure 4:**
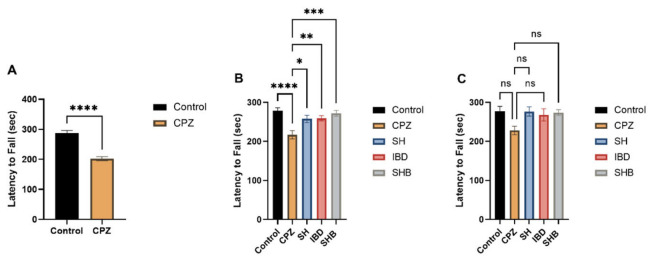
Rotarod test. (**A**) Effects of CPZ on the balance and motor coordination in the rotarod test during the demyelination stage. (**B**,**C**) Effects of different treatments on balance and motor coordination in the rotarod test during early and late remyelination stages, respectively. Data are presented as mean ± SEM; a *t*-test was used in (**A**), and one-way ANOVA followed by Tukey’s test was used in (**B**,**C**). CPZ: cuprizone, SH: 6-shogaol, IBD: Ibudilast, the combination of SHB: combination 6-shogaol and Ibudilast, ns: nonsignificant. * *p* < 0.05 ** *p* < 0.01, *** *p* < 0.001, **** *p* < 0.0001.

Compared with control mice, CPZ-treated mice exhibited a significant reduction in latency to fall on the rotarod during demyelination (*p* < 0.0001; Figure 4A) and early remyelination (*p* < 0.0001). Compared with the CPZ treatment, the SH, IBD, and SHB treatments significantly improved rotarod performance during early remyelination [SH (*p* = 0.0126), IBD (*p* = 0.0087), and SHB (*p* = 0.0004); Figure 4B]. No significant differences were observed among the groups during late remyelination as shown in (Figure 4C).

#### 2.2.3. Wire Hang Test

**Figure 5:**
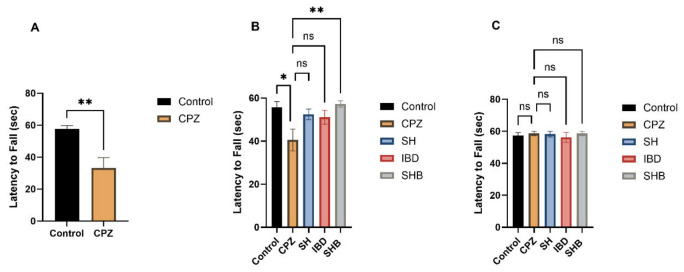
Wire hang test. (**A**) Effects of CPZ on the latency to fall during the demyelination stage. (**B**,**C**) Effects of different treatments on the latency to fall during early and late remyelination stages, respectively. Data are presented as mean ± SEM; a *t*-test was used in (**A**), and one-way ANOVA followed by Tukey’s test was used in (**B**,**C**). CPZ: cuprizone, SH: 6-shogaol, IBD: Ibudilast, SHB: combination 6-shogaol and Ibudilast ns: nonsignificant. * *p* < 0.05,. ** *p* < 0.01.

Compared with control mice, CPZ-treated mice presented significantly shorter latency to fall during demyelination and early remyelination (*p* = 0.0014 and *p* = 0.0159, respectively) (Figure 5A,B). SHB significantly improved latency during early remyelination (*p* = 0.0063). No significant differences were observed among the groups during late remyelination (Figure 5C).

#### 2.2.4. Novel Object Recognition

**Figure 6:**
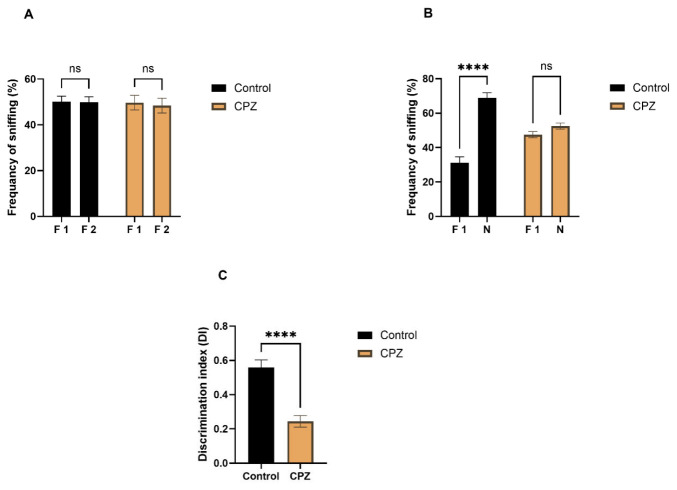
Novel object recognition test (NORT) during demyelination. (**A**) Frequency of sniffing (%) during the familiarization phase. (**B**) Frequency of sniffing (%) during the test phase. (**C**) Discrimination index (DI). Data are presented as mean ± SEM. Two-way ANOVA followed by Šídák’s post hoc test (**A**,**B**), and a *t*-test was used (**C**). F1: familiar object 1, F2: familiar object 2, N: novel object, CPZ: cuprizone, ns: nonsignificant. **** *p* < 0.0001.

At the end of demyelination, no significant difference was found between the control and CPZ groups in sniffing frequency (%) for familiar objects during the familiarization phase (*p* = 0.9379) (Figure 6A). In the test phase, the control group showed a significant preference for the novel object (*p* < 0.0001), whereas the CPZ group did not (Figure 6B). Additionally, the discrimination index (DI) was significantly lower in the CPZ group than in the control group (*p* < 0.0001) (Figure 6C).

**Figure 7:**
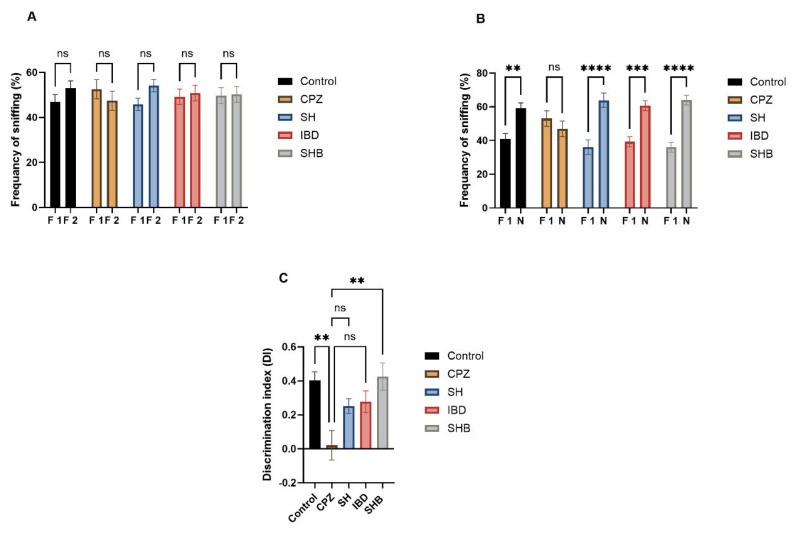
Novel object recognition test (NORT) during the late remyelination stage. (**A**) Frequency of sniffing (%) during the familiarization phase. (**B**) Frequency of sniffing (%) during the test phase. (**C**) Discrimination index (DI). Data are presented as mean ± SEM. Two-way ANOVA followed by Šídák’s post hoc test (**A**,**B**) and one-way ANOVA followed by Tukey’s post hoc test were used in (**C**). F1: familiar object 1, F2: familiar object 2, N: novel object, CPZ: cuprizone, SH: 6-shogaol, IBD: Ibudilast, SHB: combination of 6-shogaol and Ibudilast, ns: nonsiginficant. ** *p* < 0.01, *** *p* < 0.001, **** *p* < 0.0001.

During late remyelination, sniffing frequency during the familiarization phase did not differ significantly among the groups (Figure 7A). In the test phase, the control, SH, IBD, and SHB groups showed a significant preference for the novel object (*p* = 0.0056, *p* < 0.0001, *p* = 0.0009, and *p* < 0.0001, respectively), whereas the CPZ group did not (Figure 7B). DI was significantly lower in the CPZ group than in the control group (*p* = 0.0057). However, SHB treatment significantly improved the DI relative to that of CPZ (*p* = 0.0032), whereas no significant improvement was observed in the SH or IBD groups (Figure 7C).

### 2.3. Gene Expression

#### 2.3.1. NF-κB p65

**Figure 8:**
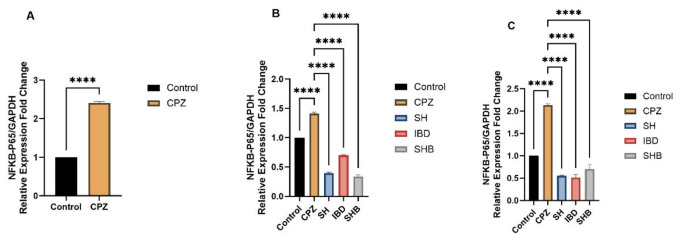
Expression level of NF-κB p65. (**A**) effects of CPZ on the expression level of NF-κB p65 during the demyelination stage. (**B**,**C**) effects of different treatments on the NF-κB p65 expression level during early and late remyelination stages, respectively. Data are presented as mean ± SEM; a *t*-test was used in (**A**), and one-way ANOVA followed by Tukey’s test was used in (**B**,**C**). NF-κB p65: Nuclear factor kappa-light-chain-enhancer of activated B cells, GAPDH: glyceraldehyde 3-phosphate dehydrogenase, CPZ: cuprizone, SH: 6-shogaol, IBD: Ibudilast, SHB: 6-shogaol + Ibudilast. **** *p* < 0.0001.

During demyelination, NF-κB p65 mRNA expression was significantly elevated in the CPZ group compared with the control group (*p* < 0.0001) (Figure 8A). The elevated expression persisted throughout both the early and late remyelination stages (*p* < 0.0001). In contrast, the SH, IBD, and SHB treatments significantly reduced NF-κB p65 expression compared with that in the CPZ group at both remyelination stages (*p* < 0.0001 for all) (Figure 8B,C).

#### 2.3.2. TNF-α

**Figure 9:**
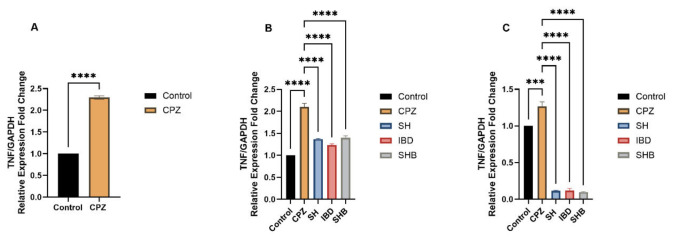
Expression level of TNFα. (**A**) effects of CPZ on the expression level of TNFα during the demyelination stage. (**B**,**C**) effects of different treatments on the TNFα expression level during early and late remyelination stages, respectively. Data are presented as mean ± SEM; a *t*-test was used in (**A**), and one-way ANOVA followed by Tukey’s test was used in (**B**,**C**). TNFα: Tumor necrosis factor, GAPDH: glyceraldehyde 3-phosphate dehydrogenase, CPZ: cuprizone, SH: 6-shogaol, IBD: Ibudilast, SHB: 6-shogaol + Ibudilast. *** *p* < 0.001, **** *p* < 0.0001.

During demyelination, TNF-α mRNA expression was significantly greater in the CPZ group than in the control group (*p* < 0.0001) (Figure 9A). This elevated expression persisted during both the early (*p* < 0.0001) and late (*p* = 0.0006) remyelination stages. In contrast, TNF-α levels were significantly lower in all the SH, IBD, and SHB treatment groups than in the CPZ group at both remyelination stages (*p* < 0.0001 for all) (Figure 9B,C).

#### 2.3.3. COX-2

**Figure 10:**
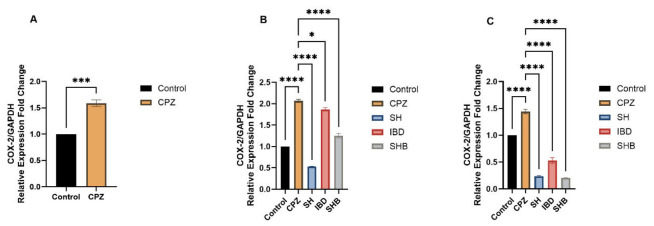
Expression level of COX-2. (**A**) effects of CPZ on the expression level of COX-2 during the demyelination stage. (**B**,**C**) effects of different treatments on the COX-2 expression level during early and late remyelination stages, respectively. Data are presented as mean ± SEM; a *t*-test was used in (**A**), and one-way ANOVA followed by Tukey’s test was used in (**B**,**C**). COX-2: Cyclooxygenase-2, GAPDH: glyceraldehyde 3-phosphate dehydrogenase, CPZ: cuprizone, SH: 6-shogaol, IBD: Ibudilast, SHB: 6-shogaol + Ibudilast. * *p* < 0.05, *** *p* < 0.001, **** *p* < 0.0001.

During demyelination, COX-2 mRNA expression was significantly elevated in the CPZ group compared with the control group (*p* = 0.0007) (Figure 10A). This increase persisted at the early remyelination stage (*p* < 0.0001). However, compared with CPZ, SH, IBD, and SHB significantly reduced COX-2 levels [SH (*p* < 0.0001), IBD (*p* = 0.0155), SHB (*p* < 0.0001)] (Figure 10B). At the late remyelination stage, COX-2 expression remained significantly greater in CPZ-treated mice than in control mice (*p* < 0.0001), whereas compared with those in CPZ-treated control mice, the levels of COX-2 expression in SH, IBD, and SHB-treated mice were significantly lower (*p* < 0.0001) (Figure 10C).

#### 2.3.4. IL-4

**Figure 11:**
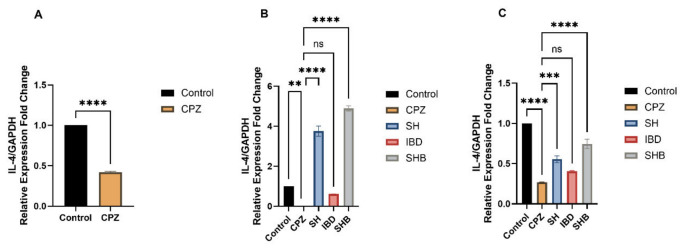
Expression level of IL-4. (**A**) effects of CPZ on the expression level of IL-4 during the demyelination stage. (**B**,**C**) effects of different treatments on the IL-4 expression level during early and late remyelination stages, respectively. Data are presented as mean ± SEM; a *t*-test was used in (**A**), and one-way ANOVA followed by Tukey’s test was used in (**B**,**C**). IL-4: Interleukin-4, GAPDH: glyceraldehyde 3-phosphate dehydrogenase, CPZ: cuprizone, SH: 6-shogaol, IBD: Ibudilast, SHB: 6-shogaol + Ibudilast ns: nonsignificant ** *p* < 0.01, *** *p* < 0.001, **** *p* < 0.0001.

During demyelination, IL-4 mRNA levels were significantly lower in the CPZ group than in the control group (*p* < 0.0001) (Figure 11A). This reduction persisted during the early (*p* = 0.0022) and late (*p* < 0.0001) remyelination stages. In contrast, IL-4 expression was significantly greater in the SH and SHB groups than in the CPZ group at the early stage (*p* < 0.0001). A similar pattern was observed in the late stage, with SH (*p* = 0.0007) and SHB (*p* < 0.0001) showing higher IL-4 levels than CPZ. No significant differences were found between the IBD and CPZ groups at either remyelination stage (Figure 11B,C).

#### 2.3.5. NRF2

**Figure 12:**
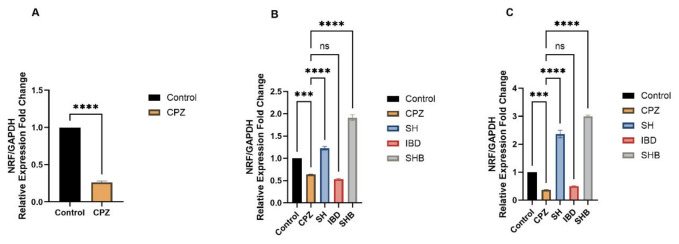
Expression level of Nrf2. (**A**) effects of CPZ on the expression level of Nrf2 during the demyelination stage. (**B**,**C**) effects of different treatments on the Nrf2 expression level during early and late remyelination stages, respectively. Data are presented as mean ± SEM; a *t*-test was used in (**A**), and one-way ANOVA followed by Tukey’s test was used in (**B**,**C**). Nrf2: Nuclear factor erythroid 2–related factor 2, GAPDH: glyceraldehyde 3-phosphate dehydrogenase, CPZ: cuprizone, SH: 6-shogaol, IBD: Ibudilast, SHB: 6-shogaol + Ibudilast ns: nonsignificant. *** *p* < 0.001, **** *p* < 0.0001.

During demyelination, Nrf2 expression was significantly lower in the CPZ group than in the control group (*p* < 0.0001; Figure 12A). Similarly, Nrf2 levels remained significantly suppressed in the CPZ group during both the early and late remyelination stages (*p* = 0.0002). In contrast, the SH and SHB groups presented significant upregulation of Nrf2 expression compared with the CPZ group at both time points (*p* < 0.0001). However, no significant differences in Nrf2 expression were observed between the IBD and CPZ groups during either remyelination stage (Figure 12B,C).

## 3. Discussion

This study aimed to address the fact that, to date, no approved MS medications target remyelination, which remains a critical therapeutic goal [24]. In this context, CPZ was utilized as a copper chelator that induces demyelination by generating reactive oxygen species (ROS), causing apoptosis in mature oligodendrocytes [25]. CPZ is a highly reproducible model suitable for evaluating therapies that support myelin repair [15,16]. It also reflects behavioral symptoms associated with demyelinating diseases, such as motor impairment, memory loss, and mood changes [26,27]. Most previous studies used young adult mice (6–9 weeks old) fed a diet containing 0.2–0.3% CPZ for 5–6 weeks to induce acute demyelination [16]. Furthermore, several factors, including sex, body weight, and mouse strain, influence susceptibility to CPZ intoxication [28,29], which were carefully controlled in the present study.

Our findings indicate that the combined treatment leads to notable improvements in motor coordination, neuromuscular strength and memory performance, accompanied by significant modulation of key inflammatory and anti-inflammatory gene expression. To our knowledge, this is the first study to investigate the effects of SH and IBD in this model, introducing a promising therapeutic approach that aligns with recent literature advocating for combinatorial strategies to address the multifactorial pathology of MS [30].

Behavioral analyses provided clear evidence for functional improvement with SH, IBD, and their combination (SHB) during the remyelination phase following CPZ-induced demyelination. In the open field test (OFT), CPZ-treated mice exhibited significant reductions in total distance moved and velocity, indicating motor suppression and reduced exploratory activity, which is consistent with demyelination [25,26]. Treatment with SH and IBD independently improved behavioral impairments, whereas SHB produced the most significant improvement, suggesting a functional additive effect in restoring locomotor activity [20,21]. Similar patterns were observed in the rotarod test, where CPZ exposure significantly reduced the latency to fall, reflecting deficits in motor coordination and balance [31,32]. SH and IBD partially restored performance, whereas SHB significantly improved latency values toward control levels during early remyelination, underscoring its therapeutic superiority. In the wire hang test, only SHB significantly improved neuromuscular strength, unlike SH or IBD alone, indicating superior neuromuscular recovery with the combination therapy [33]. Finally, in the novel object recognition test (NORT), CPZ exposure disrupted recognition memory, as shown by a decreased discrimination index (DI) [34,35]. SHB markedly reversed these cognitive deficits, whereas SH and IBD alone had limited effects. These behavioral outcomes likely stem from the complementary molecular actions of SH and IBD. Crucially, the superior performance of the SHB combination in restoring recognition memory and neuromuscular strength suggests that their mechanisms, including NF-κB inhibition and Nrf2 activation, contribute to the observed improvement in efficacy and functional recovery. This functional superiority aligns with recent findings demonstrating that dual-targeting strategies enhance functional recovery and myelin repair in CNS disorders [36]. Collectively, these findings suggest that SHB promotes functional recovery across multiple behavioral domains relevant to MS pathology.

At the molecular level, CPZ-induced demyelination led to increased expression of proinflammatory genes, including NF-κB-p65, TNF-α, and COX-2, which is consistent with previous findings linking these mediators to oligodendrocyte damage and microglial activation in MS [37,38,39]. Treatment with SH significantly reduced the expression of NF-κB, COX-2, and TNF-α, supporting its role as a potent anti-inflammatory agent that inhibits the NF-κB pathway and downstream cytokine production [40,41]. IBD also reduced NF-κB and TNF-α expression, which is consistent with its ability to increase intracellular cAMP and suppress glial-mediated inflammation [20,42]. Specifically, IBD acts as a non-selective phosphodiesterase inhibitor, primarily targeting PDE4 to elevate cAMP/PKA signaling, which in turn deactivates microglial inflammatory cascades. Its effect on COX-2 was less pronounced, which may point to a different downstream target. Notably, SHB combination therapy resulted in the most marked reduction in the expression of all three genes during both the early and late remyelination phases, suggesting a significant improvement in efficacy in modulating neuroinflammatory signaling. The complementary actions of SH, which primarily inhibits ROS-induced NF-κB activation, and IBD, which moderates cytokine signaling via PDE inhibition, likely underlie the superior neuroprotective responses observed with SHB. Overall, these results support the view that combining SH and IBD offers broader protection against inflammation-related demyelination in experimental models of MS.

In contrast to the proinflammatory milieu induced by CPZ, the expression of IL-4 and Nrf2 was significantly suppressed in demyelinated mice, reflecting a compromised endogenous protective response [43,44]. This suppression indicates that CPZ not only triggers inflammation but also weakens intrinsic antioxidant capacity, thereby amplifying oxidative damage. SH treatment effectively restored IL-4 levels and strongly upregulated Nrf2 expression, highlighting its dual role in promoting a Th2-skewed immune response and enhancing resistance to oxidative stress [40,45,46], consistent with previous evidence supporting the neuroprotective role of ginger-derived compounds in neurodegenerative diseases [47]. These effects are thought to involve the regulation of the NF-κB pathway and the activation of the Nrf2–HO-1 axis, both of which are established molecular targets of SH [47,48]. Notably, 6-shogaol is recognized as one of the most potent Nrf2 activators among ginger constituents, effectively counteracting the oxidative stress that impairs OPC maturation [49]. Conversely, IBD did not significantly influence IL-4 or Nrf2 expression, indicating a more confined anti-inflammatory mechanism centered on PDE inhibition and glial modulation rather than direct transcriptional activation of cytoprotective pathways [50]. Thus, the inclusion of SH was essential to provide the necessary Nrf2-mediated antioxidant defense, which IBD alone could not elicit. Notably, the SHB combination produced the greatest upregulation of both IL-4 and Nrf2, exceeding the effects of SH alone. This synergistic upregulation highlights the added value of combining both agents. Taken together, the coactivation of IL-4 and Nrf2 by SHB demonstrates its capacity to simultaneously attenuate neuroinflammation, promote neuroprotection, and support myelin repair.

Comparative evaluation of the treatment groups revealed that SH and IBD exert beneficial effects through distinct yet complementary mechanisms that converge when combined in the SHB regimen. SH has broader effects by modulating behavior, suppressing inflammation, and activating antioxidant defenses. Its ability to restore IL-4 and Nrf2 expression positions it as a multifaceted agent with both immunomodulatory and neuroprotective properties [45,46,47]. Conversely, the therapeutic action of IBD appears to be more focused on reducing glial-driven neuroinflammation through PDE inhibition and cAMP elevation, leading to consistent downregulation of NF-κB and TNF-α with limited engagement of antioxidant pathways [20,42,50]. This combined effect reflects the multifactorial nature of MS pathology and supports the rationale for combinatorial approaches targeting diverse aspects of disease progression.

While our findings demonstrate significant functional and molecular improvements with the combination of 6-shogaol and ibudilast, we acknowledge key limitations. The selected doses were based on previously reported effective ranges, and therefore the enhanced outcomes observed in the combination group may be partly expected. Accordingly, the observed effect is more appropriately described as a functional additive effect rather than a validated pharmacological synergy. Moreover, pharmacodynamic analyses, dose–response evaluation, and formal synergy assessments (e.g., isobolographic analysis or Combination Index (CI) calculations) were not performed. While this study provides robust functional and molecular evidence supporting the therapeutic potential of the SH and IBD combination, future investigations incorporating protein-level validation and direct histological assessment of myelin repair would further strengthen these findings. Additionally, given the acute and non-autoimmune nature of the cuprizone model, validation in autoimmune experimental models would broaden the translational relevance of these results.

In conclusion, our findings demonstrate that the combination of 6-shogaol and Ibudilast (SHB) offers enhanced therapeutic potential compared with either agent alone in a CPZ-induced mouse model of MS. SHB is uniquely effective in restoring behavioral function, suppressing proinflammatory signaling, and enhancing anti-inflammatory and antioxidant gene expression. These effects highlight the importance of targeting multiple pathological mechanisms, including oxidative stress and glial activation, to promote myelin repair and neuroprotection. Overall, the integration of SH-mediated Nrf2 activation and IBD-mediated glial regulation yields a complementary effect that may help address the multifactorial nature of MS, supporting its potential as a candidate for further preclinical investigation in more clinically relevant autoimmune models of MS.

In accordance with the ARRIVE 2.0 guidelines, this study ensured clear and comprehensive reporting of the experimental design, including group allocation, outcome measures, and statistical analyses. Adherence to these guidelines enhances the transparency, reproducibility, and reliability of the findings.

## 4. Materials and Methods

### 4.1. Animals and Ethics

Seventy-five male SWR/J mice (6–8 weeks old, 18–21 g) were obtained from the King Fahd Medical Research Center (King Abdulaziz University, Jeddah, Saudi Arabia). The mice were housed 3–5 per cage under standard conditions (12 h light/dark cycle, 23 ± 2 °C), with free access to food and water. All experimental procedures were approved by the Biomedical Ethics Committee of KAU (Ref No. 617-20) and the Animal Care and Use Committee at King Fahd Medical Research Center (Protocol Code: ACUC-20-10-24) and were conducted in accordance with the National Institutes of Health Guide for the Care and Use of Laboratory Animals. All experimental procedures were also designed and reported in accordance with ARRIVE 2.0 guidelines, following the recommendations of the EQUATOR network to ensure transparency and reproducibility.

Male mice were exclusively used to minimize variability associated with hormonal fluctuations, which may influence neuroinflammatory responses and behavioral outcomes. The experimental protocol was well-tolerated, and no mortality was recorded throughout the study (0% mortality rate).

### 4.2. Drugs

#### 4.2.1. Cuprizone

Cuprizone (CPZ; C9012-25G, Thermo Fisher Scientific, Waltham, MA, USA) was mixed with chow at 0.3% (*w*/*w*) and administered for 5 weeks to induce acute demyelination [51].

#### 4.2.2. 6-Shogaol

6-shogaol (SH; CAS No. 555-66-8; Cat. No. BP0095) was purchased from BioPurify Phytochemicals Ltd. (Chengdu, China). It was dissolved in saline containing 5% dimethyl sulfoxide (DMSO) and administered intraperitoneally (i.p.) at a dosage of 25 mg/kg/day, based on previous evidence supporting the neuroprotective and anti-inflammatory effects of 6-shogaol in experimental MS and other neuroinflammatory models [22,52].

#### 4.2.3. Ibudilast

IBD (Cat. No. A11019-2000) was purchased from Adooq Bioscience (Irvine, CA, USA). The solution was dissolved in 5% DMSO (in normal saline) and administered i.p. at a dose of 10 mg/kg/day, which has been validated for its efficacy in modulating glial activation and neuroinflammation [20,53].

#### 4.2.4. Rationale for the Combined 6-Shogaol and Ibudilast Treatment

The combined treatment regimen was selected based on the previously reported therapeutic efficacy of 6-shogaol and ibudilast as individual agents in experimental models of neuroinflammation and demyelination. The dose of 6-shogaol was chosen because of its documented anti-inflammatory and antioxidant properties, particularly its ability to suppress NF-κB-mediated inflammatory signaling and activate Nrf2-associated cytoprotective pathways. Ibudilast was selected because it is a phosphodiesterase inhibitor with established anti-inflammatory and neuroprotective effects, including suppression of glial activation and reduction in pro-inflammatory cytokine production. Since cuprizone-induced demyelination involves oxidative stress, glial activation, inflammatory cytokine release, and impaired functional recovery, the combination of 6-shogaol and ibudilast was designed to provide a multi-target therapeutic approach. The doses used in the combination group were the same as those administered in the respective monotherapy groups, allowing comparison of the effects of each compound alone and in combination.

### 4.3. Experimental Design

**Figure 13:**
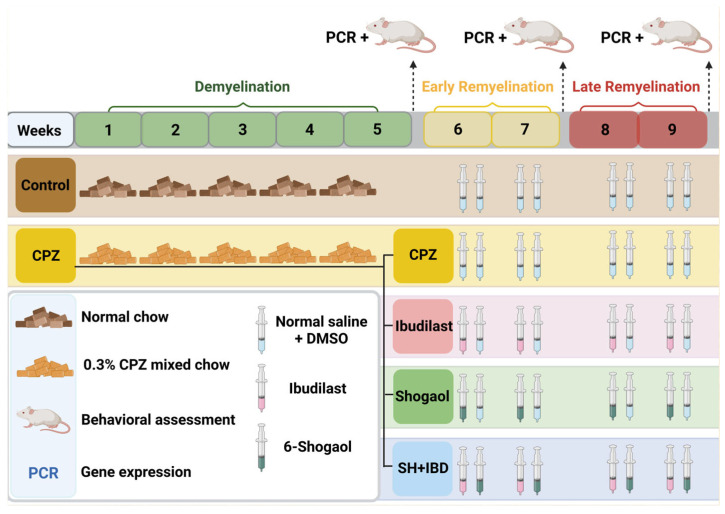
Timescale of the study. The total duration of the study was 9 weeks. Mice were exposed to 0.3% CPZ for 5 weeks to induce demyelination, followed by 4 weeks of remyelination. During the remyelination phase, the CPZ group was subdivided into four treatment groups: CPZ-only (vehicle-treated), SH (6-shogaol, 25 mg/kg/day), IBD (ibudilast, 10 mg/kg/day), and SHB (combination of 6-shogaol and ibudilast, 25 mg/kg and 10 mg/kg, respectively). Behavioral tasks and quantitative measurement of gene expression were determined by the end of weeks 5,7 and 9. Created in BioRender. ALSHAHRANY, G. (2026) https://BioRender.com/90oas7v (accessed on 23 June 2026).

The experiment was conducted over 9 weeks: during the first 5 weeks, demyelination was induced using CPZ. Mice were initially divided into two groups: a control group (*n* = 18) and a CPZ-treated group (*n* = 57), with randomization performed while maintaining equal mean body weights across groups to minimize potential confounding effects. At the end of week 5, CPZ was withdrawn and five animals from each group were sacrificed for tissue collection and molecular analyses, resulting in *n* = 13 in the control group and *n* = 52 in the CPZ group, which were then subdivided into four treatment groups (*n* = 13 each): CPZ-only (vehicle-treated), SH-treated, IBD-treated, and SHB (combination-treated), for the 4-week remyelination phase. This phase was further divided into early remyelination (week 7) and late remyelination (week 9), with four animals per group sacrificed at the end of each sub-phase for tissue collection, resulting in *n* = 9 per group at week 7 and *n* = 5 per group at week 9. Animals were sacrificed at each stage of the experiment in order to obtain tissue samples for molecular analyses. Mice were placed in a plexiglass chamber with 5% Isoflurane (Baxter Healthcare, Deerfield, IL, USA) and decapitated when they were totally anesthetized. Immediate head dissection was performed using sharp, small tools to extract the brain on a cold surface while maintaining tissue integrity. The collected brains were preserved in RNAlater^®^ solution (Thermofisher Scientific, CAT. # AM7024) for gene expression analysis. All treatments were administered daily between 1:00 and 3:00 PM. Each mouse received two daily intraperitoneal (i.p.) injections: one containing the active compound (or vehicle) and a second containing saline with residual DMSO, such that all groups, including the control, were exposed to an equivalent total DMSO concentration. Behavioral assessments and gene expression analysis were performed at the end of each experimental stage, as illustrated in Figure 13. The sample size was determined based on previously published studies using the cuprizone model, with a total of 75 animals used throughout the study.

### 4.4. Body Weight Assessment

Mouse weight (g) was measured at each study time point. The percentage of weight gain was calculated via the following formula: weight gain% = [(final weight − initial weight)/initial weight] × 100.

### 4.5. Behavioral Assessments

Behavioral assessments were performed at the end of weeks 5, 7, and 9, with *n* = 13 in the control group and *n* = 52 in the CPZ group (*n* = 13 per each subgroup) at week 5, *n* = 9 per group at week 7, and *n* = 5 per group at week 9, Animals were sacrificed at each stage of the experiment in order to obtain tissue samples for molecular analyses. Anxiety-like behavior was assessed using the open field test prior to behavioral testing as a predefined exclusion criterion; no animals met the exclusion threshold and all were retained for subsequent analyses. Animals were habituated to the testing room for 30 min before each session and were handled daily for one week prior to testing to reduce stress-related confounds. To minimize bias, all assessments and data analyses were performed by team members blinded to group allocation.

#### 4.5.1. Open Field Test (OFT)

Locomotor activity was assessed via the OFT. Each mouse was placed in a 45 × 45 cm arena and allowed to explore freely for 3 min [54]. The total distance moved (TDM) and velocity (cm/s) were automatically tracked and recorded via the EthoVision XT8A system (Noldus Information Technology, Wageningen, The Netherlands).

#### 4.5.2. Rotarod

Motor coordination and balance were evaluated via an accelerating rotarod (4–40 rpm over 5 min). Following habituation, each mouse underwent three trials with 90 min rest intervals, and the latency to fall (in seconds) was recorded and averaged for analysis [55].

#### 4.5.3. Wire Hang Test

Forelimb neuromuscular strength was assessed via the use of a 40 cm metal wire suspended above a padded surface. The mice were allowed to grip the wire with their forelimbs, and latency to fall (maximum 60 s) was recorded across three trials. The average latency was used as the final score [34].

#### 4.5.4. Novel Object Recognition Test (NORT)

Recognition memory was assessed via the NORT as previously described [54]. The test spanned two days: on day one (habituation), the mice explored a 45 × 45 cm empty arena for 3 min. On day two, during familiarization, the mice explored two identical objects for 3 min. After a 10 min interval, one object was replaced with a novel object differing in shape and color, and the mice were allowed to explore again for 3 min. The arena and objects were cleaned with 10% ethanol after each trial. Sniffing frequency (%) was calculated as (sniffing of novel or familiar object/total sniffing) × 100. Recognition memory was also measured by the discrimination index: (time with novel − time with familiar)/ (time with novel + time with familiar). Behavioral data were recorded and analyzed via the EthoVision XT8A system [56].

### 4.6. Quantitative Real-Time PCR (qRT–PCR)

RNA was extracted from 100 mg of brain tissue via TRIzol (Invitrogen, Carlsbad, CA, USA), purified with chloroform, isopropanol, and 75% ethanol, and dissolved in RNase-free water. The RNA concentration and purity were assessed via a Nanodrop ND-1000, and the samples were stored at −80 °C [57]. cDNA was synthesized from 500 ng of RNA via SuperScript™ IV with random hexamers and dNTPs (Invitrogen, USA). qPCR was performed on a StepOne™ Real-Time PCR System (Applied Biosystems, USA) with PowerUp™ SYBR Green Master Mix (Applied Biosystems, Foster City, CA, USA) to quantify NF-κB p65, TNFα, COX-2, IL-4, and Nrf2. Each 10 µL reaction contained 5 µL of master mix, 1 µL of each primer (10 pmol), and 3 µL of diluted cDNA template (100 ng/µL). The samples were cycled at 50 °C for 2 min and 95 °C for 2 min, followed by 45 cycles of 95 °C for 5 s and 60 °C for 10 s. The reactions were run in triplicate, and gene expression was analyzed via the 2−ΔΔCT method and normalized to that of GAPDH [58]. The primer sequences are listed in Table 1.

### 4.7. Statistical Analysis

The data were analyzed via GraphPad Prism 9.5.1 and are presented as the means ± SEMs. One-way ANOVA followed by Tukey’s post hoc test was used for all results during the early and late remyelination stages, except for the NORT sniffing frequency in late remyelination, where two-way ANOVA with Šídák’s post hoc test was applied. Unpaired *t* tests were used for demyelination data. A *p* value < 0.05 was considered statistically significant. Analyses were conducted separately at each experimental stage to avoid violating independence assumptions across time points. Statistical reporting in this study follows the SAMPL guidelines.

## Figures and Tables

**Table 1:** PCR primer sequences.

Gene	Oligo	Sequences
GAPDH	Forward primer	AGGTCGGTGTGAACGGATTTG
	Reverse primer	GGGGTCGTTGATGGCAACA
NF-κB p65	Forward primer	CGGGATGGCTACTATGAGGCTGAC
	Reverse primer	GATTCGCTGGCTAATGGCTTGCT
TNF-α	Forward primer	GGTCCCCAAAGGGATGAGAAGT
	Reverse primer	TTGCTACGACGTGGGCTAC
COX-2	Forward primer	TGAGTACCGCAAACGCTTCT
	Reverse primer	CAGCCATTTCCTTCTCTCCTGT
IL-4	Forward primer	CTCACAGCAACGAAGAACACCA
	Reverse primer	CTTCAAGCATGGAGTTTTCCCA
Nrf2	Forward primer	TGTAGATGACCATGAGTCGCTTG
	Reverse primer	TATTGAGGGACTGGGCCTGAT

## Data Availability

The datasets generated and analyzed during the current study are available from the corresponding author upon reasonable request due to privacy restrictions.
